# Point-of-care detection of lipoarabinomannan (LAM) in urine for diagnosis of HIV-associated tuberculosis: a state of the art review

**DOI:** 10.1186/1471-2334-12-103

**Published:** 2012-04-26

**Authors:** Stephen D Lawn

**Affiliations:** 1 The Desmond Tutu HIV Centre, Institute for Infectious Disease and Molecular Medicine, Faculty of Health Sciences, University of Cape Town, Anzio Road, Observatory, 7925, Cape Town, South Africa; 2 Department of Clinical Research, Faculty of Infectious and Tropical Diseases, London School of Hygiene and Tropical Medicine, London, UK

**Keywords:** HIV, Tuberculosis, Screening, Diagnosis, Culture, Asymptomatic, Subclinical

## Abstract

Detection of *Mycobacterium tuberculosis* antigens in urine is attractive as a potential means of diagnosing tuberculosis (TB) regardless of the anatomical site of disease. The most promising candidate antigen is the cell wall lipopolysaccharide antigen lipoarabinomannan (LAM), which has been used to develop commercially available enzyme-linked immunosorbent assays. Although highly variable diagnostic accuracy has been observed in different clinical populations, it is now clear that this assay has useful sensitivity for diagnosis of HIV-associated TB in patients with advanced immunodeficiency and low CD4 cell counts. Thus, this assay is particularly useful when selectively used among patients enrolling in antiretroviral treatment services or in HIV-infected patients requiring admission to hospital medical wards. These are the very patients who have the highest mortality risk and who stand to gain the most from rapid diagnosis, permitting immediate initiation of TB treatment. A recently developed low-cost, lateral-flow (urine ‘dip-stick’) format of the assay provides a result within 30 minutes and is potentially a major step forward as it can be used at the point-of-care, making the possibility of immediate diagnosis and treatment a reality. This paper discusses the likely utility of this point-of-care assay and how it might best be used in combination with other diagnostic assays for TB. The many further research studies that are needed on this assay are described. Consideration is particularly given to potential reasons for the variable specificity observed in existing field evaluations of LAM ELISAs. Whether this might be related to the assay itself or to the challenges associated with study design is discussed.

## Review

### Introduction

Tuberculosis (TB) remains a major challenge to global health [1]. There were an estimated 8.8 million incident cases in 2010 and, of 1.45 million TB deaths, 0.35 million were in HIV-positive people [2]. The HIV-associated TB epidemic has been one of the major stumbling blocks to TB control, accounting for an estimated 12.5% of the global TB caseload in 2010. A large majority (82%) of these cases were concentrated in sub-Saharan Africa where HIV has had an extraordinary impact on TB incidence rates and TB-related mortality [2,3].

Despite the fact that low-income and middle-income countries account for over 90% of the worldwide burden of TB, these regions still rely heavily on sputum smear microscopy and chest radiology for TB diagnosis [1,4]. These techniques often perform poorly and are typically unavailable at patients’ first point of contact with the health system. Moreover, the diagnostic accuracy of these techniques is substantially impaired in those with HIV coinfection [4,5]. Although simple point-of-care assays are available and routinely used for diagnosis of HIV infection and malaria [6], this is not the case for TB. There is a huge need for rapid point-of-care tests for TB with high diagnostic accuracy that can be readily used at all levels of the health system and in the community.

### Urine antigen testing for TB diagnosis

*Mycobacterium tuberculosis* antigen detection has long been viewed positively as an option for TB diagnosis as this has the potential advantage of reflecting mycobacterial burden while remaining unimpaired by immune status. Moreover, analysis of urine rather than sputum samples is a very attractive option as urine is simple to collect without generating hazardous bioaerosols, it is safe to handle in the laboratory, it has relatively few bacterial contaminants and sample quality is unlikely to be highly variable.

A number of mycobacterial antigens can be detected in the urine of patients with pulmonary TB [7,8], but the most promising of these to emerge is the cell wall lipopolysaccharide lipoarabinomannan (LAM) [9-11]. Enzyme-linked immunosorbent assays (ELISAs) that detect LAM have been commercially available as TB diagnostic assays for a number of years [9]. More recently a simple, low-cost lateral flow version of this assay has been developed and the first clinical evaluations have been published [12,13]. This paper reviews the utility of assays for urinary LAM for diagnosing HIV-associated TB in adults and discusses the potential of the lateral-flow LAM assay. Rigorous field evaluations of this assay are needed and we discuss the associated challenges in study design.

### What is lipoarabinomannan (LAM)?

**Figure 1:**
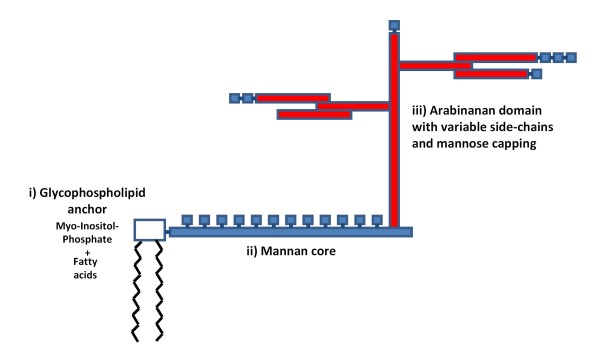
Cartoon showing the basic structure of mycobacterial lipoarabinomannan (LAM) and its three main domains. (i) The glycophospholipid anchor binds the molecule to the plasma membrane of the organism. (ii) The mannan core is attached to this and is highly conserved across mycobacterial species. (iii) The variable branching arabinan side chains and the variable mannose capping of these side chains gives rise to the diversity of LAM molecules.

LAM is one of three major groups of interrelated lipopolysaccharides within the mycobacterial cell wall [14-16]. All these molecules are non-covalently attached to the mycobacterial plasma membrane via the glyco-phospholipid anchor and extend to the surface of the cell wall. LAM molecules have three major structural domains (Figure 1). The phospholipid anchor is linked to a carbohydrate (mannose) core, which is conserved across all mycobacterial species, and from this carbohydrate (arabinofuranosyl) side-chains arise [14]. Variable capping of the arabinosyl side-chains with mannose residues results in a diversity of LAM molecules with a range of unique properties and functions. The presence of mannose capping allows mycobacteria to bind to mannose receptors on macrophages, which provide the preferred intracellular environment for the organism [14].

The pattern of capping characterises these LAM molecules as belonging to one of three major classes. LAM molecules with mannosylated caps on the terminal D-arabinan side chains (ManLAM) are characteristic of more pathogenic mycobacterial species such as *M. tuberculosisMycobacterium leprae* and *Mycobacterium bovis*[14]. These molecules serve as major virulence factors, causing inhibition of a range of processes including phagosome maturation, apoptosis, interferon-γ-signalling in macrophages and interleukin-12 secretion by dendritic cells and also serving as a scavenger for toxic oxygen free radicals [14,17]. Thus, LAM plays an important role in promoting survival of *M. tuberculosis* in the human host.

While ManLAMs are found in pathogenic species, LAM capped with phosphoinositol (so-called PILAMs) are typically found in non-pathogenic species such as *Mycobacterium smegmatis*[14]. Other species of rapidly growing mycobacteria such as *Mycobacterium chelonae* contain LAMs have no mannose or phosphoinositol capping and are termed AraLAM molecules. AraLAM and PILAM have a strong proinflammatory effect within the human host whereas ManLAM molecules have potent immunomodulatory properties.

LAM from any particular source is heterogeneous with regard to size, the pattern of branching of the side-chains and acylation and phosphorylation of the mannan core and the arabinan side chains [14]. The peak molecular weight has been found to be centered at 17.3 kDa, but with a broad distribution either side, reflecting considerable molecular heterogeneity [18]. This diversity of the LAM family of molecules is of importance with regard to the sensitivity and specificity of immunoassays for LAM. LAM is heat-stable and so does not readily degrade in clinical samples, increasing its suitability as a diagnostic target.

### How does LAM enter the urine?

LAM can be recovered in large quantities from cultures of *M. tuberculosis* (15 mg per gram of bacteria) in vitro [16] and there is some evidence to suggest that LAM is actively secreted from infected alveolar macrophages [17]. Such an active process would be consistent with the important immunomodulatory properties of LAM that are likely to favour survival of the organism *in vivo*. Development of high tissue concentrations of LAM at anatomic sites of disease may favour entry of the antigen into the systemic circulation such that LAM can be detected in serum samples from patients with pulmonary TB [19]. Systemic antigenaemia may also result directly from dissemination of *M. tuberculosis* in the blood stream, especially in patients with advanced HIV-associated immunodeficiency [20].

The mechanism whereby LAM enters the urine from the systemic circulation is unclear. LAM has a similar molecular size to myoglobin, which readily passes into urine from the bloodstream following release from damaged muscles in people with normal renal glomerular function [21]. Thus, it is possible that free LAM molecules might similarly enter urine in this way. However, LAM is antigenic, eliciting a strong humoral antibody response [22] and so it is likely that LAM largely exists within the circulation in the form of circulating immune complexes which would not readily cross the glomerular basement membrane. The proportions of complexed and uncomplexed LAM in blood may be an important determinant of LAM-antigenuria and thus deficiencies in the humoral immune responses to LAM could theoretically be an important determinant of antigenuria. Similarly, whether LAM within urine is antibody-bound may also be important as LAM within immune complexes may be difficult for immunoassays to detect due to steric hindrance.

It has been postulated that renal dysfunction (such as HIV nephropathy) may underlie the ability of LAM to enter the urine from the bloodstream. A study from Tanzania found that detection of any protein in urine samples by simple urinalysis stick tests was associated with LAM antigenuria [23] whereas similar testing in a study in South Africa found no such association [24]. A more careful analysis using laboratory quantification of urine protein excretion found that successful detection of LAM in the urine of patients with TB was not associated with heavy proteinuria that would be indicative of severe glomerular dysfunction that would be required to permit free filtration of immune-complexed LAM [25]. This would therefore suggest that this is not an important factor.

Recent studies of patients with HIV-associated TB and LAM detectable in the urine have found that, even using small urine aliquots, approximately one half of these urine samples test positive by Xpert MTB/RIF assay [25,26]. In contrast, LAM-negative samples from TB patients all tested Xpert MTB/RIF-negative [25]. Since Xpert MTB/RIF detects whole *M. tuberculosis* bacilli, positive results using this assay indicate the presence of *M. tuberculosis* in the renal tract of these patients.

In conclusion, it seems possible that free circulating LAM could enter the urine readily, but not if present in large immune complexes. LAM antigenuria, however, could also potentially result from direct involvement of the renal tract with TB such that antigen may enter the urinary tract directly without passing across the renal glomerular basement membrane.

### Clinical evaluation of commercial ELISAs

Early studies demonstrated the ability to detect LAM in the sputum, serum and urine from patients with TB [19,27-29], giving moderate diagnostic sensitivity and specificity. However, these assays required extensive sample preparation, which included column chromatography, rendering them impractical for routine clinical diagnosis. However, with proof of principle data from a simplified polyclonal sandwich ELISA test that required much less intensive sample processing, the first ELISA was released onto the commercial market [30]. This was initially produced by Chemogen Inc. as the ‘MTB ELISA’ test and was subsequently marketed as ‘Clearview TB ELISA’ by Inverness Medical Innovations and latterly by Alere Inc. Sample preparation for these assays requires heating the urine sample to 95–100^0^ C for 30 minutes and then centrifugation at 10,000 rpm for 15 minutes at room temperature. The supernatant is then tested in a 96 well plate format ELISA.

Nine clinical evaluations of the commercially produced LAM ELISAs have been published to date using sputum culture as the gold standard [12,23,24,30-35] (Table 1). Of these, 5 were done in South Africa and the remainder in Tanzania (two studies), Zimbabwe and India. The first of these studies published in 2005 evaluated the Chemogen assay in Tanzania and found a moderately high sensitivity (74–81%) and very high specificity (99%) both among HIV-infected and non-infected ambulatory patients with pulmonary TB [30]. No study has since replicated these data and the reasons for this remain unclear.

**Table 1:** Studies evaluating commercially available assays detecting urinary lipoarabinomannan (LAM) for diagnosis of tuberculosis (TB) in patients with culture-confirmed disease

**Study**	**LAM assay**	**Country**	**Study population**	**Total subjects (n)**	**HIV+ subjects with culture+ TB (n)**	**HIV- subjects with culture+ TB (n)**	**Culture method**	**Sensitivity of sputum microscopy % (95%CI)**	**Sensitivity of LAM assays for culture+ TB % (95%CI)**		**Overall specificity % (95%CI)**	**LAM results and non-tuberculous mycobacteria**	
									**HIV+ subjects**	**HIV- subjects**		**% subjects with NTM who tested LAM+**	**% false- positive LAM with NTM cultured**
Boehme et al. 2005 [30]	MTB ELISA, first prototype (Chemogen Inc)	Tanzania	Out-patient TB suspects	334	85	34	LJ culture	62 (54-70)	81 (71-88)	74 (57-86)	99 (94-100)	NS	NS
Lawn et al. 2009 [31]	MTB ELISA, second prototype (Chemogen Inc)	South Africa	Active screening of HIV+ out-patients pre-ART	235	58	0	Automated liquid culture	14 (7-25)	All: 33 (22-46)	N/A	100 (98-100)	None	None
									CD4>100: 13 (4-33)				
									CD4 50-100: 41 (22-64)				
									CD4<50: 67 (44-84)				
Mutetwa et al. 2009 [32]	MTB ELISA, second prototype (Chemogen Inc)	Zimbabwe	Out-patient TB suspects	397	140	Not stated	LJ culture	75 (67-81)	52 (43-62)	21 (9-37)	89 (81-94)	NS	NS
Reither et al. 2009 [23]	MTB ELISA second prototype (Chemogen Inc)	Tanzania	Out-patient TB suspects	291	50	19	LJ and automated liquid culture	70 (58-79)	62 (48-74)	21 (8-44)	88 (79-94)	4/45 (8.9%)	4/14 (29%)
Daley et al. 2009 [33]	MTB ELISA, second prototype (Chemogen Inc)	India	Out-patient TB suspects	200	5	40	LJ and liquid culture	79* (66-89)	20 (1-70)	18 (8-32)	88 (81-92)	NS	NS
Shah et al. 2009 [34]	Clearview TB ELISA (Inverness Medical Innovations)	South Africa	In-patient HIV+ TB suspects	499	167	14	Automated liquid culture	42 (36-50)	All: 67 (59-74)	14 (3-41)	96 (91-99)	NS	NS
									CD4>200 55 (41-69)				
									CD4150-200: 14 (4-58)				
									CD4150-200: 56 (30-80)				
									CD450-100: 71 (51-87)				
									CD4<50: 85 (73-93)				
Dheda et al. 2010 [24]	Clearview TB ELISA (Inverness Medical Innovations)	South Africa	Out-patient TB suspects	500	44	80	Automated liquid culture	65 (57-72)	All: 21 (11-35)	6 (3-14)	99 (97-100)	NS	NS
									CD4<200: 37 (19-59)				
Gounder et al 2011 [35]	Clearview TB ELISA (Inverness Medical Innovations)	South Africa	Active screening of HIV+ out-patients pre-ART or on ART	422	30	0	Automated liquid culture	27 (12-48)	All: 32 (16-52)	N/A	98 (96-99)	4/7 (57%)	4/8 (50%)
									CD4200-350: 10 (1-72)				
									CD4<200: 35 (16-57)				
									CD4<50: 56 (21-86)				
Lawn et al. 2011 [12]	Clearview TB ELISA (Alere Inc.)	South Africa	Active screening of HIV+ out-patients pre-ART	516	85	0	Automated liquid culture	28 (19-39)	All: 27 (18-38)	N/A	98 (96-99)	2/8 (25%)	2/8 (25%)
									CD4>200: 8 (1-26				
									CD4<200: 36 (24-49)				
									CD4<150: 44 (29-59)				
									CD4<100: 48 (29-68)				
									CD4<50 61 (36-83)				
Lawn et al 2012 [12]	Determine TB-LAM Ag point-of-care test	South Africa	Active screening of HIV+ out-patients pre-ART	516	85	0	Automated liquid culture	28 (19-39)	All: 28 (19-39)	N/A	99 (97-100)	2/8 (25%)	2/6 (33%)
									CD4>200: 4 (0-20)				
									CD4<200: 39 (27-53)				
									CD4<150: 46 (31-61)				
									CD4<100: 52(33-71)				
									CD4<50: 67 (41-87)				
Peter et ak. 2012 [13]	Determine TB-LAM Ag point-of-care test	South Africa	In-patient HIV+ TB suspects	281 TB suspects + 88 non-TB controls	116	0	Automated liquid culture	56 (45-66)	Multiple different analyses using different gold standards and different assay cut-offs: see text for summary	N/A	See text	NS	NS

*of all TB diagnoses, not just culture-confirmed. 95%CI = 95% confidence intervals. Where 95%CI were not calculated in the original manuscripts, these have been calculated using the Wald method.

LJ = Lowenstein Jensen slopes. ELISA = enzyme-linked immunosorbent assay. ART = antiretroviral therapy. NS: not stated.

**Figure 2:**
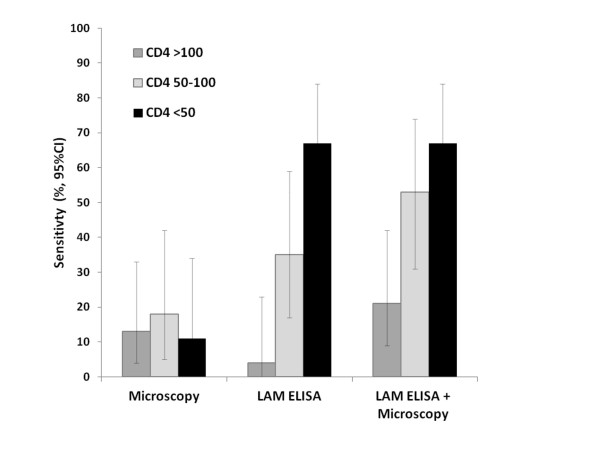
Graph showing the sensitivity of a commercially available enzyme-linked immunosorbent assay (ELISA) to detect lipoarabinomannan (LAM) within urine samples to diagnose tuberculosis (TB) in a cohort of patients accessing antiretroviral treatment (ART) in a South African township. The sensitivity (%) of sputum microscopy and the LAM ELISA are shown individually and combined (either positive) compared with a gold standard of automated liquid culture of two sputum samples. Data are stratified by CD4 cell count (cells/μL) and show that the sensitivity of the LAM ELISA was substantially greater in the patients with the lowest CD4 cell counts. Figure reproduced from Lawn et al. 2011 [5] and data originally from Lawn et al. 2009 [31].

Subsequent evaluations in HIV-noninfected patients have found the assay sensitivity for sputum culture-positive TB to be very poor indeed (6%–21%) (Table 1). In contrast, the sensitivity among HIV-infected TB patients has been found to be greater (21%–67%). A key observation is that sensitivity is very strongly related to blood CD4 cell count, with higher sensitivity observed at lower CD4 cell counts (Figure 2). Among patients with CD4 cell counts <50 cells/μL, sensitivity has ranged between 56% and 85% (Table 1), greatly exceeding the sensitivity of sputum smear microscopy. When results of microscopy and the LAM ELISA are combined (either test positive), there is incremental diagnostic sensitivity (Figure 2). This indicates that among patients with sputum culture-positive TB, microscopy and LAM diagnose different sub-groups of individuals.

Reported specificity also varies between the 9 studies (Table 1). Six of these studies (including all 5 from South Africa) have found specificity to be 96%–100%. In contrast, studies from Tanzania, Zimbabwe and India reported specificities of 88%–89%. The issue of variable specificity between studies is very important and the range of potential contributing factors is considered in detail later in this article.

### Development of a point-of-care LAM assay

**Figure 3:**
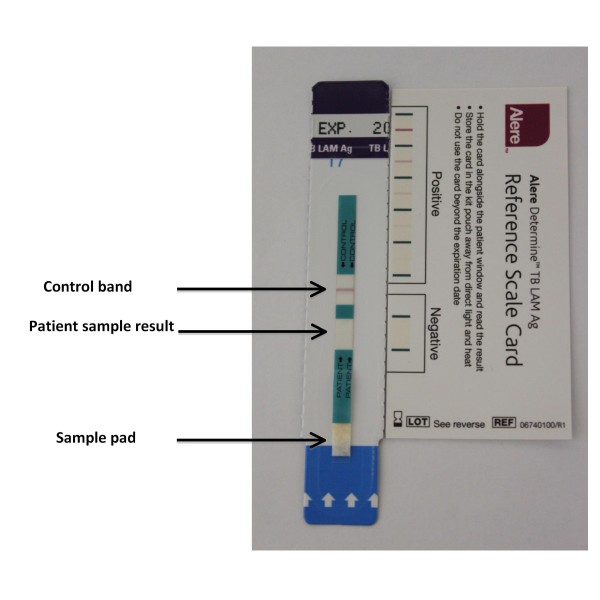
Photograph of a Determine TB-LAM test strip showing the sample pad to which 60 μL of the test urine is applied. After 25–35 minutes of incubation at room temperature, the control band is checked and the sample test result is read. Reading may be facilitated by comparison to the reference card. The presence of a band in the sample window of similar or greater intensity to the weakest positive on the reference card indicates the presence of lipoarabinomannan (LAM) in the urine.

The commercially available LAM ELISAs are 96-well plate format sandwich assays that employ highly purified polyclonal antibody preparations. This format of the assay is not suitable for use in resource-limited settings due to infrastructure limitations and the need for sample preparation and batch processing in centralised laboratories. However, using the same antibody preparations, a simple lateral-flow version of the assay has been produced as a point-of-care test (Figure 3). Determine TB-LAM Ag ([Determine TB-LAM] Alere, Waltham, MA, USA) is an immunochromatographic assay in which the capture antibodies are adsorbed onto the nitrocellulose membrane of the test strip and the detection antibody is labelled by conjugation to colloidal gold particles.

The assay procedure is very simple. A volume of 60 μL of unprocessed urine is added to the sample pad where colloidal gold labelled antibodies bind to LAM present in the sample (Figure 3). As the urine sample moves along the test strip, complexed LAM is then captured by the LAM antibodies immobilized in a line on the nitrocellulose membrane. The colloidal gold particles result in the development of a visible purple line, indicating the presence of LAM in the sample (Figure 3). A control bar is also included in the sample test strip to ensure assay validity. The test strip is read between 25 and 35 minutes of incubation at room temperature. The intensity of any visible band is compared with the faintest positive line on the reference card. Any line with similar or greater line intensity is scored as testing positive for LAM. Thus, the assay result is a qualitative (positive/negative) test for LAM.

### Clinical evaluation of a point-of-care LAM assay

The first clinical evaluation of the Determine TB-LAM assay was among HIV-infected patients with advanced immunodeficiency enrolling in an antiretroviral treatment (ART) service in a South African township [12]. All eligible patients were screened for TB regardless of the presence or absence of symptoms. Patients were classified as either having TB (based on a diagnostic gold-standard of culture-based detection of *M. tuberculosis* in one or more sputum samples) or not having TB (based on negative sputum cultures). The diagnostic accuracy of Determine TB-LAM was also compared Xpert MTB/RIF - a simplified rapid molecular assay that was endorsed by the World Health Organization in December 2010 as a replacement for sputum smear microscopy in resource-limited settings [36,37].

Urine samples obtained at the time of screening were stored at −20°C and retrospectively tested in a laboratory environment rather than prospectively at the point-of-care [12]. Blinded assessments of the test strips by two independent readers showed extremely high agreement (к = 0.97). Results were very similar to those obtained by the Clearview TB-ELISA run on the same samples (к = 0.84), with marginally fewer false-negative and false-positive results using the Determine TB-LAM test-strips.

**Figure 4:**
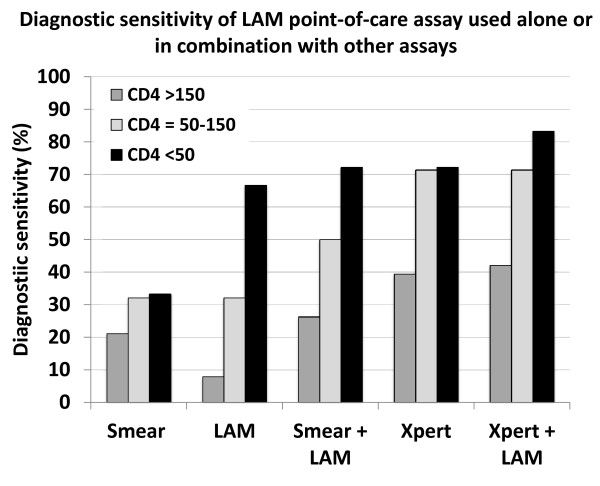
Graph showing the diagnostic sensitivity for sputum culture-positive tuberculosis using the Determine TB-LAM assay for lipoarabinomannan (LAM) when used alone or in combination with other diagnostics, including sputum smear microscopy (smear) and Xpert MTB/RIF (Xpert). Data are stratified by CD4 cell count (cells/μL) and show that the sensitivity of the LAM was substantially greater in the patients with the lowest CD4 cell counts. There was important incremental sensitivity when microscopy and Determine TB-LAM were combined and this was similar to the sensitivity of Xpert in patients with the lowest CD4 cell counts.

In those with sputum culture-positive TB (n = 85), the sensitivity of Determine TB-LAM stratified by CD4 cell count (Figure 4) [12] was remarkably similar to that observed using the Chemogen ELISA in the earlier study conducted in the same study population (Figure 2) [31]. This is reassuring in that it demonstrates substantial consistency in the performance of these LAM assays which have been sequentially derived from one another over the past 5 years. Although overall sensitivity was low (28.2%), it was highest in those with the lowest CD4 count categories (66.7% in those with CD4 counts <50 cells/μL). Moreover, there was incremental sensitivity when results of Determine TB-LAM and smear microscopy were combined (either test positive), attaining a sensitivity of 72.2% in those with CD4 cell counts <50 cells/μL. This was comparable to the sensitivity of a single sputum Xpert MTB/RIF test in this patient group (Figure 4).

Specificity of Determine TB-LAM was assessed in patients with negative sputum cultures regardless of clinical status (n = 431) and was 98.6% overall and exceeded 98% in all analyses stratified by CD4 cell count and other patient characteristics [12]. The positive predictive value (PPV) exceeded 90% in all patients with CD4 cell counts <150 cells/μL, those with a preceding advanced WHO clinical stage (3 or 4) and among those with chest radiographs that were reported as showing any abnormality (however minor) that was consistent with pulmonary TB. The negative predictive value of the assay (89.8%) was not sufficient for reliable exclusion of TB.

A second study by Peter and colleagues from South Africa has evaluated Determine TB-LAM among hospital in-patients [13]. Two groups of patients were recruited: HIV-infected patients suspected of having TB (n = 335) and a control group of HIV-infected patients with a non-TB diagnosis (n = 88). An drawback of this study compared to previous evaluations of LAM assays is that patients did not undergo standardized investigation for TB by the study team. Instead the results from investigations during routine medical care were used, with samples being processed in the routine laboratory system.

Data were analysed in a number of different ways. One key finding was that when the faintest positive bands on the test strips were used (grade 1 cut-point) as recommended by the manufacturer, specificity in culture-negative patients was very poor (66%, 95%CI, 57–74) but improved to 96% (95%CI, 89–100) using the grade 2 cut-point [13]. Inter-observer agreement was also higher using the grade 2 cut-point. This finding differs from that of Lawn and colleagues [12] and this raises the possibility of variation between different manufacturing lots of the test strips. No data have yet been published examining batch to batch variation. Another possible explanation might be bacterial contamination of urine samples, giving rise to weak false-positive bands as discussed later in this article.

Sensitivity was assessed using two different analyses, first using a culture-based gold-standard and, second, a gold standard that additionally incorporated clinico-radiological diagnoses with a positive response to TB treatment [13]. Data were not stratified by CD4 cell count, but overall Determine TB-LAM had a sensitivity of 45% compared to the composite gold standard and 71% when results were combined with those of sputum smear microscopy.

### Potential utility of determine TB-LAM point-of-care assay

The attractive features of Determine TB-LAM are that it can be used at the point-of-care within an out-patient clinic setting or on a hospital ward. Thus, patients can be rapidly diagnosed (30 minutes per test) and treated at a single clinic visit. It is low-cost (approximately $3.50 per test) compared to approximately $18 per cartridge for an Xpert MTB/RIF assay or over $20 for automated liquid culture. The assay can be used by health care workers with no laboratory training and without need for any diagnostic hard-ware or electricity supply. It does not generate large volumes of biohazardous waste and the test strips can be stored at room temperature.

The assay only has utility in those with HIV infection and advanced immunodeficiency (CD4 cell counts <200 cells/μL and/or WHO stage 3 or 4 disease) and therefore should not be used to test unselected TB suspects attending medical out-patient clinics or TB clinics. Instead, the assay should be targeted for use in those with known HIV infection being screened for TB prior to starting ART or requiring admission to hospital medical in-patient wards. Since the assay sensitivity is so strongly associated with blood CD4 cell count, it would best be used in patients with CD4 cells counts known to be <200 cells/μL. This is a draw-back for resource-limited settings where CD4 count testing may not routinely be available.

Determine TB-LAM would ideally be used in combination with other diagnostic assays, providing incremental diagnostic sensitivity and/or expediting diagnosis to permit TB treatment to be started at a single clinic visit. The potential advantages of various combinations of assays are shown in Table 2. Smear microscopy alone is the mostly widely available testing strategy in resource-limited settings and use of Determine TB-LAM in combination would increase diagnostic sensitivity without great additional cost (Figure 4). This would permit rapid diagnosis in those with the most advanced immunodeficiency and highest mortality risk.

**Table 2:** Utility of Determine TB-LAM point-of-care assay when used in combination with other diagnostic tests for HIV-associated tuberculosis (TB)

**Combination of diagnostic tests**	**Advantages**
Sputum smear microscopy + Determine TB-LAM	· Incremental sensitivity.
	· Smear microscopy diagnoses most infectious patients
	· Determine TB-LAM provides rapid point-of-care diagnosis in those with high mortality risk permitting immediate initiation of TB treatment.
	· Low-cost.
Chest radiology + Determine TB-LAM	· Determine TB-LAM can provide rapid point-of-care assessment of patients with abnormal chest radiographs, increasing the specificity for TB diagnosis.
	· Small incremental cost to radiology.
Sputum culture + Determine TB-LAM	· Determine TB-LAM greatly expedites TB diagnosis in the sickest patients with highest mortality risk.
	· Small incremental cost to culture.
Sputum Xpert MTB/RIF + Determine TB-LAM	· Determine TB-LAM adds little diagnostic sensitivity when used with Xpert MTB/RIF, but provides rapid point-of-care assessment. When Xpert is located in laboratories precluding same-day testing, Determine TB-LAM will expedite TB treatment in those with highest mortality risk, allowing immediate initiation of TB treatment.
	· Small incremental cost to Xpert MTB/RIF.

Chest radiography permits rapid patient evaluation, but the radiological features of HIV-associated TB in those with CD4 cell counts <200 cells/μL are often very non-specific [4,38] and the differential diagnosis can be difficult and time-consuming to resolve. However, the positive predictive value of Determine TB-LAM for TB was found to be high among those with radiographic abnormalities [12] and thus combined testing would increase diagnostic specificity in those with abnormal radiographs.

Mycobacterial culture is available in very few resource-limited settings, but is invaluable for accurate diagnosis of smear-negative TB. The very long time to culture-based diagnosis in such patients (3 weeks in a study in South Africa [31]) is a major draw-back, however, and this delay would be greatly reduced in a proportion of the patients with highest mortality risk by combined use of Determine TB-LAM screening

In some countries such as South Africa, the Xpert MTB/RIF assay is being implemented nationwide to replace sputum smear microscopy as the first diagnostic assay. Determine TB-LAM provides little incremental sensitivity when used in combination with Xpert MTB/RIF [12] (Figure 4), and yet this combination may have an important advantage over use of Xpert MTB/RIF in isolation. Due to feasibility, cost and logistical issues, the Xpert MTB/RIF is likely to be implemented in centralised laboratories, leading to a critical disconnect between the patient and the test results. This is problematic. In a study in Cape Town, South Africa, where Xpert MTB/RIF is being rolled out in centralised laboratories, only 76.6% of patients diagnosed as having Xpert-positive TB during pre-ART screening started TB treatment [26,39]. Moreover, these patients only started TB treatment after a median delay of 9 days (interquartile range, 6–18 days) [26]. This delay was due to an overall turnaround time of 4 days for the sample to reach the laboratory, be processed, and for the results to be issued and reach the clinic. Additional delays accrued waiting for patients to return following recall; others were found to have already died or were lost to follow-up. Determine TB-LAM could close this treatment gap for the patients who have the lowest CD4 cell counts and highest mortality risk, permitting TB treatment initiation at the first clinic visit.

There is currently much interest in development of strategies to reduce early mortality in ART programmes in sub-Saharan Africa [40], and TB is recognised as one of the key causes of death [41]. In the absence of suitable diagnostics, trials have been designed to evaluate a strategy whereby HIV-infected patients in high TB prevalence settings with very low CD4 counts but no overt TB diagnosis are randomised to receive or not receive empiric TB treatment, with mortality being the primary outcome of interest [42]. These are multicentre trials are being conducted in sub-Saharan Africa and include the AIDS Clinical Trial Group (ACTG) REMEMBER trial (NCT01380080) and the PROMPT study (NCT01417988) funded by the European Developing Countries Clinical Trials Programme (EDCTP). However, the recent emergence of the Determine TB-LAM assay has to some extent undermined the rationale for such studies in view of its ability to rapidly detect those with TB and high mortality risk.

### Further research

Many more studies on the Determine TB-LAM assay are needed and a list of research questions that need to be addressed is given in Table 3. These questions fall into three main groups: 1) those related to gaining a better understanding of the assay, improving its performance and investigating optimum sample storage; 2) those assessing diagnostic accuracy and issues surrounding implementation, and 3) those assessing impact and cost. A robust evidence base is needed to permit public health policy development with regards to implementation of this assay.

**Table 3:** Further research that is needed regarding the Determine TB-LAM assay

**Focus of research**	**Goal**	**Studies needed**
Assay and samples	Understanding the assay	Determine mechanisms by which LAM enters urine. Further clarify the host and pathogen factors associated with LAM antigenuria
	Assess any batch to batch variation of the assay	To determine manufacturing quality control of the test strips
	Improve diagnostic accuracy	Research and development to identify means to improve sensitivity and specificity Studies to assess and enhance reproducibility of interpreting test strips
	Assess impact of conditions of sample storage	Determine the effect of duration of urine storage at room temperature and any impact of freeze-thaw cycles on diagnostic accuracy
	Assess impact of urine contamination	Assess impact of contamination of urine samples on specificity
Diagnostic accuracy and assay utility	Assess diagnostic accuracy	More studies are needed to very carefully assess the sensitivity and specificity in different geographical locations in appropriate clinical populations (advanced HIV, ambulatory vs hospitalized, adults vs children) using appropriate diagnostic gold standard. Assess for any association between reduced specificity and non-tuberculous mycobacteria or other coinfections/co-morbidity
	Use by health care workers at point-of-care	Assess feasibility and acceptability of running and reading the test-strips at point-of-care by non-laboratory trained health-care workers
	Incorporation in diagnostic algorithms	Operational research to assess feasibility and utility of incorporating in different diagnostic algorithms
Impact and cost	Impact assessment	Assess the impact on time to diagnosis, time to starting TB treatment, morbidity and mortality and programme efficiency
	Cost-effectiveness	Assess cost-effectiveness in different settings

### Assay specificity: a key unresolved issue

Studies have consistently reported that the LAM assays (ELISAs or point-of-care assay) have useful sensitivity in HIV-infected patients with low CD4 cell counts [12,24,31,34]. On this point, there is consensus. However, the issue of assay specificity is not so clearly defined. Studies done within South Africa have all found specificity to lie in the range 96% to 100% [12,24,31,34,35]. In contrast, studies from Tanzania, Zimbabwe and India reported specificities of 88%–89% [23,32,33]. The reasons for this have not been identified although factors that might have contributed in part might be proposed. In the Zimbabwe study, for example, solid rather than liquid culture was used which may have limited the diagnostic sensitivity of the gold standard [32]. Another of the studies investigated predominantly HIV-noninfected patients, which is an non-recommended (‘off-label’) indication for the assay [33]. Despite this, however, low specificity was also seen in the HIV-infected sub-groups included in the Tanzania and Zimbabwe studies [23,32].

There are a range of possible reasons for low specificity in some studies:

i) Sensitivity of the gold standard assay for TB diagnosis. The best gold standard diagnostic assay for TB is liquid culture, which has greater sensitivity than solid media culture. However, the sensitivity of any culture method may also vary between laboratories depending, for example, on the intensity of the sputum decontamination procedure, the supplements added to the growth media and other variables. Thus, over-decontamination of sputum samples, for example, may lead to apparent false-positive LAM-antigenuria.

ii) Reliance on sputum samples for gold standard diagnosis. All studies to date have relied on sputum culture as the diagnostic gold standard except for the study by Shah and colleagues which also included mycobacterial blood cultures [34]. This is potentially problematic for several reasons. Not all cases of HIV-associated TB have pulmonary involvement yielding positive respiratory samples [43]. Thus, patients with HIV-associated TB may have negative sputum cultures but nevertheless have LAM antigenuria. Also, poor quality sputum samples may cause failure of TB detection in patients who may nevertheless have LAM antigenuria. Obtaining respiratory samples of sufficient quality may be especially challenging in very sick hospital in-patients. In such patients, cough strength may be impaired and infection control hazards in the ward environment may mitigate against good sputum collection. In this regard, specificity might be expected to appear lower in studies of in-patients rather than ambulatory out-patients. Use of routine sputum induction done on all study participants in an appropriate clinical setting is likely to be needed as was done in studies in a South African ART service [12,31,44]. The microbiological gold-standard would be considerably enhanced by examining both pulmonary and extrapulmonary samples. Fine needle aspirates of enlarged lymph nodes and urine samples [25,43,45,46] might also be examined using culture or the Xpert MTB/RIF assay and mycobacterial blood cultures might be done to provide a composite microbiological gold standard. This, would make studies somewhat more intensive both from clinical and laboratory stand points.

iii) Use of clinical follow-up. The use of clinical follow-up for suspecting or refuting a TB diagnosis in the presence of negative microbiology is problematic in HIV-infected patients with advanced immunodeficiency. Clinical follow-up may not be possible due to death, the risk of which is high in this patient population. Early ART initiation may alter the clinical picture. In addition, a high rate of new incident TB and frequent co-pathology often confuses the clinical picture.

iv) Non-tuberculous mycobacteria. A key question is whether non-tuberculous mycobacterial species in vivo could cause LAM-antigenuria. As discussed earlier, LAM encompasses large family of related molecules which are expressed by all mycobacterial species. Although *M. tuberculosis* strains were the most strongly reactive, pre-clinical studies *in vitro* did find cross-reactivity between a range of mycobacterial strains (including *Mycobacterium fortuitum, Mycobacterium kansasii, and Mycobacterium bovis*) when using the prototype ELISA developed by Chemogen [30]. Similarly, a LAM ELISA system reported by Kadival and colleagues also showed low-level cross reactivity with *Mycobacterium avium, M. kansasii and M. fortuitum*[47]. Thus, this is a possible reason for lowered specificity of LAM assays and might explain differences between studies in different countries as the prevalence of these organisms has substantial geographical variation. In Table 1, the detection of non-tuberculous mycobacteria during diagnostic accuracy studies is shown but this was reported in just 4 of the 9 studies. The proportion of false-positive LAM results that was associated with culture of non-tuberculous mycobacteria from sputum ranged from zero [31] to 4 out of 8 (50%) [35]. Of note, in the study from Tanzania in which specificity of the Chemogen MTB ELISA was just 88%, 4 of 14 (29%) false positives were associated with culture of non-tuberculous mycobacteria. Whether these associations are causal (ie associated with invasive non-tuberculous mycobacterial disease and LAM-antigenuria) or whether these organisms are simply colonisers of the airway or sputum contaminants is not yet known. Non-mannosylated LAM molecules that are more typical of non-tuberculous mycobacteria are very strongly proinflammatory. Thus, it might be expected that these forms of LAM would not be present in the systemic circulation in sufficient concentrations in an uncomplexed form that could then result in detectable antigenuria. Careful research on this issue is needed.

v) Urine sample contamination. It is known that the anti-LAM antibodies used in the commercial LAM assays cross-react with a number of non-mycobacterial species. Dheda and colleagues reported that cultures containing common oral flora such as various species of Actinobacteria (strains of Nocardia and Streptomyces) and Candida showed reactivity in the Clearview TB ELISA [24]. It is not inconceivable that the presence of similarly reactive species on the perineum could contaminate urine samples and lead to false-positive tests, especially if samples were left for periods at room temperature permitting replication of these organisms. Similarly, it is likely to be important that urine is collected and stored in sterile containers.

## Conclusions

The Determine TB-LAM assay is the first point-of-care assay for TB and this has been found to have specific utility in those with advanced HIV-associated immunodeficiency. These are the very patients who could potentially gain the most from rapid point-of-care diagnosis, permitting initiation of TB treatment at a single clinic visit. The first clinical evaluation in the field [12] was very encouraging although a second study reported more difficulties in reading of the test strips [13]. There is a strong rationale to push ahead with many more studies. These should further evaluate diagnostic accuracy in a range of geographical settings; assess utility and feasibility in in-patient and ambulatory out-patient clinical populations, including children; determine how best to incorporate the assay within diagnostic algorithms and assess cost and impact. This assay might potentially have been discarded on the basis of apparent sub-optimal sensitivity. However, with studies in appropriate clinical populations providing new insights into the real utility of this assay, the assay is now emerging as an important tool that may serve to greatly expedite TB diagnosis and treatment in those with advanced HIV-associated immunodeficiency, potentially reducing mortality risk.

## Competing interest

The author has no conflicts of interest what so ever to declare.

## Pre-publication history

The pre-publication history for this paper can be accessed here:

http://www.biomedcentral.com/1471-2334/12/103/prepub
